# Non-linear association of birth weight with lung function and risk of asthma: A population-based study

**DOI:** 10.3389/fpubh.2022.999602

**Published:** 2022-11-24

**Authors:** Meng Yang, Hong Mei, Juan Du, Linling Yu, Liqin Hu, Han Xiao

**Affiliations:** ^1^Institute of Maternal and Child Health, Wuhan Maternal and Child Health Care Hospital, Tongji Medical College, Huazhong University of Science and Technology, Wuhan, China; ^2^Department of Occupational and Environmental Health, School of Public Health, Tongji Medical College, Huazhong University of Science and Technology, Wuhan, China

**Keywords:** lung function, asthma, threshold effect, birth weight, public health

## Abstract

**Background:**

The impact of birth weight on lung function and risk of asthma remains contentious. Our aim was to investigate the specific association of birth weight with lung function and the risk of asthma in children.

**Methods:**

We performed cross-sectional analyses of 3,295 children aged 6–15 years who participated in the 2007–2012 National Health and Nutrition Examination Survey (NHANES). After controlling for potential covariates other than gestational diabetes, maternal asthma and obesity, the linear and non-linear associations of birth weight with lung function metrics and the risk of asthma were evaluated by a generalized linear model and generalized additive model, respectively.

**Results:**

We observed a non-linear association of birth weight with FEV_1_ %predicted, FEV_1_/FVC %predicted and FEF_25 − 75_ %predicted (*P* for non-linearity was 0.0069, 0.0057, and 0.0027, respectively). Further threshold effect analysis of birth weight on lung function detected the turning point for birth weight was 3.6 kg. When the birth weight was < 3.6 kg, birth weight was significantly positively associated with all pulmonary function metrics. However, negative associations were found in FEV_1_ %predicted, FEV_1_/FVC %predicted and FEF_25 − 75_ %predicted when the birth weight was ≥3.6 kg. These results were consistent in the stratified and sensitivity analyses. Additionally, a possible non-linear relationship was also detected between birth weight and the risk of asthma.

**Conclusion:**

Although not all maternal factors were accounted for, our findings provided new insight into the association of birth weight with lung function. Future studies are warranted to confirm the present findings and understand the clinical significance.

## Background

Convincing evidence supports the notion that factors affecting intrauterine and early life could influence subsequent health and disease in later childhood and adulthood (1). Among these factors, birth weight is recognized as an important indicator of intrauterine development (2). Epidemiological studies have suggested that there may be associations between birth weight and a wide range of health outcomes, ranging from metabolic diseases, cardiovascular diseases and cardiovascular risk factors, various cancers, and respiratory diseases (3–5). As for respiratory health, numerous studies have shown that birth weight was positively correlated with children's lung function—children with lower birth weight have poorer lung function (6, 7). However, most of the previous studies focus on the adverse effects of low birth weight and the linear relationship between birth weight and lung function (8, 9). Furthermore, results about the association of birth weight with the risk of asthma are conflicting (10, 11). Considering the influence of many factors on health outcomes is not a simple linear relationship but exists the phenomenon of a threshold effect, the specific association of birth weight with lung function and asthma warrants further exploration.

In the current study, we used the generalized linear models and generalized additive models to investigate the relationship between birth weight and lung function during childhood in a large sample that was representative of the U.S. population. Moreover, we also evaluated whether birth weight was associated with the risk of asthma.

## Methods

### Study population

**Figure 1:**
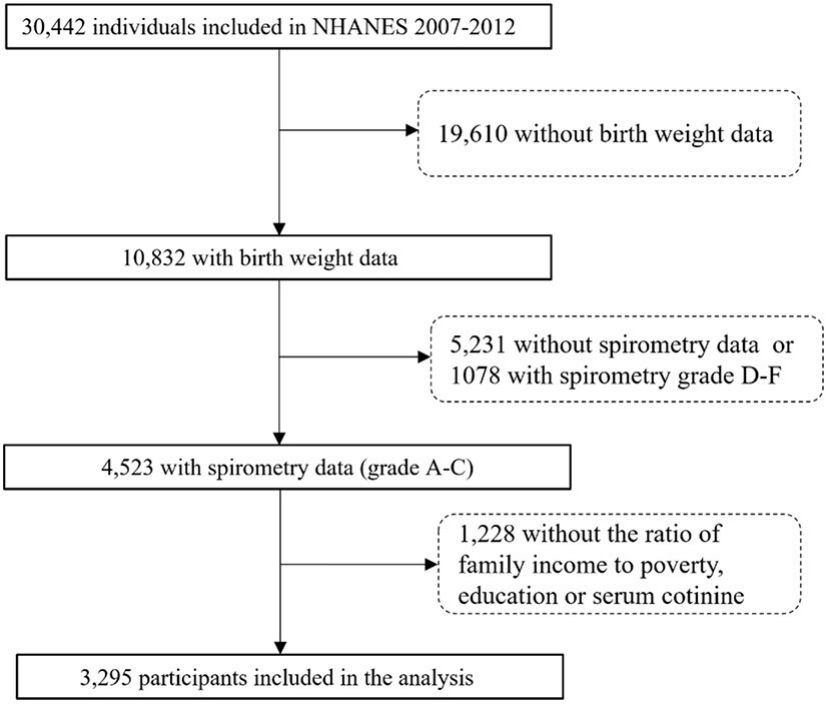
Flow diagram of the study population.

The NHANES is designed by the National Center for Health Statistics (NCHS) of the Centers for Disease Control and Prevention, which applies a complex sampling frame to attain a sample representative of the United States population (12). We collected publicly available data for 30,442 participants assessed during the 2007–2012 survey cycles. A total of 4,523 participants were aged 6–15 years consisting of birth weight and eligible spirometry data. Following exclusions for missing data on interviews or exams, 3,295 participants were included in our final analysis (Figure 1). Interviews of children aged 6–11 were conducted accompanied by the assistance of an adult familiar with the situation of children. Participants aged 12–15 answered by themselves. The NHANES was approved by the ethics review board of the National Center for Health Statistics of the Centers for Disease Control and Prevention (CDC). All participants provided written informed consent and the study was performed in accordance with ethical standards of supervising institutional review boards of all centers involved.

### Birth weight

Participants aged birth to 15 years were eligible for the birth weight *via* the NHANES Early Childhood Questionnaire (ECQ). Birth weight was recorded in pounds and ounces as reported by the adult proxy and later converted to kilograms (kg). Fetal macrosomia was defined as birth weight >4 kg (13).

### Lung function measurements

Participants aged 6 and older were eligible for spirometry examination in the NHANES 2007–2012, which is a routinely used clinical pulmonary function test. A standardized protocol was used to assess lung function according to the recommendations of the American Thoracic Society (ATS). Participants were required to repeat the test until the spirogram was acceptable and reproducible. Then the three best spirometry readings were rated A-F in line with ATS criteria and recorded (14). In this study, we focused our analysis on four pulmonary function metrics, which consisted of forced expiratory volume in one second (FEV_1_), forced vital capacity (FVC), FEV_1_/FVC and forced expiratory flow between 25 and 75% (FEF_25 − 75_), and only participants with FEV_1_ and FVC values grade A to C were included. The above four spirometric values were converted to %predicted according to the Global Lung Function Initiative 2012 equations (15) or NHANES III equations (16), which consider the participants' age, gender, height, and race/ethnicity.

### Covariates

All covariates included in the models were selected *a priori* according to prior empirical evidence. The participant information on age, gender, race/ethnicity, education level, family income, mother's age at birth of children and mother's smoking status during pregnancy was self-reported *via* interview questionnaires. The ratio of family income to poverty (PIR) was calculated by dividing family income by the poverty guidelines. PIR is a measure of socioeconomic status and >1 means at or above the poverty level (17). Current asthma status was defined by respondents giving a positive response to one question: “Has a doctor or other health professional ever told you that you had asthma?.”

### Statistical analysis

The descriptive statistical analysis summarized demographic characteristics and lung function according to birth weight quartiles. To investigate the association of birth weight with lung function, our statistical analysis consisted of three main steps. Step 1: We used a generalized linear regression model to assess the association of birth weight with lung function, including a crude model and an adjusted model with all covariates presented in Table 1. Step 2: We conducted a generalized additive model to test non-linearity using penalized smoothing regression splines with a degree of 3. If non-linearity was detected, we first calculated the turning point using likelihood-ratio tests and then constructed a 2-piecewise model on both sides of the turning point. We determined the best fit model (1-line linear regression model vs. piecewise model) based on the *P*-values for the log-likelihood ratio test. Step 3: The subgroup analyses were performed using the generalized linear regression model. Tests for effect modification by subgroup were based on interaction terms between subgroup indicators. Also, we performed the same analysis in step 1 and 2 to assess the relationship between birth weight and the risk of asthma.

**Table 1:** Characteristics of participants aged 6–15 in NHANES 2007–2012 by birth weight (*n* = 3,295).

**Characteristics**	**Total**	**Birth weight quartiles [Median (IQR)]**			
		**Q1 (< 2.95kg)** **2.72 (2.35–2.86)**	**Q2 (2.95–3.32kg)** **3.18 (3.06–3.23)**	**Q3 (3.32–3.66Kg)** **3.48 (3.40–3.57)**	**Q4 (>3.66Kg)** **3.91 (3.80–4.20)**
No. subjects	3295	843	827	822	803
Age, year (mean ± SD)	10.48 (2.77)	10.39 (2.78)	10.51 (2.69)	10.48 (2.82)	10.55 (2.80)
Height, cm (mean ± SD)	146.10 (17.02)	144.02 (16.42)	145.95 (16.74)	146.26 (17.49)	148.28 (17.22)
**Race/ethnicity (%)**					
Mexican American	823 (25.0)	193 (22.9)	223 (27.0)	199 (24.2)	208 (25.9)
Other Hispanic	389 (11.8)	93 (11.0)	103 (12.5)	102 (12.4)	91 (11.3)
Non-Hispanic White	986 (29.9)	208 (24.7)	227 (27.4)	260 (31.6)	291 (36.2)
Non-Hispanic Black	797 (24.2)	271 (32.1)	190 (23.0)	182 (22.1)	154 (19.2)
Other race-including multi-racial	300 (9.1)	78 (9.3)	84 (10.2)	79 (9.6)	59 (7.3)
**Gender (%)**					
Boys	1692 (51.4)	362 (42.9)	403 (48.7)	449 (54.6)	478 (59.5)
Girls	1603 (48.6)	481 (57.1)	424 (51.3)	373 (45.4)	325 (40.5)
**Education levels (%)**					
≤ 5 grade	2150 (65.3)	563 (66.8)	542 (65.5)	529 (64.4)	516 (64.3)
6–8 grade	911 (27.6)	221 (26.2)	234 (28.3)	231 (28.1)	225 (28.0)
9–12 grade, No Diploma	234 (7.1)	59 (7.0)	51 (6.2)	62 (7.5)	62 (7.7)
Mother's age at birth of children, year (mean ± SD)	26.39 (6.18)	26.12 (6.50)	26.19 (6.07)	26.29 (6.12)	26.99 (5.99)
**Maternal smoking during pregnancy (%)**					
Yes	412 (12.5)	156 (18.5)	106 (12.8)	78 (9.5)	72 (9.0)
No	2883 (87.5)	687 (81.5)	721 (87.2)	744 (90.5)	731 (91.0)
**Poverty index ratio (PIR) (%)**					
PIR ≤ 1	1106 (33.6)	304 (36.1)	290 (35.1)	282 (34.3)	230 (28.6)
PIR>1	2189 (66.4)	539 (63.9)	537 (64.9)	540 (65.7)	573 (71.4)
**Asthma (%)**					
Yes	613 (18.6)	167 (19.8)	157 (19.0)	133 (16.2)	156 (19.4)
No	2682 (81.4)	676 (80.2)	670 (81.0)	689 (83.8)	647 (80.6)
**NHANES cycles**					
2007–2008	967(29.3)	234 (27.8)	256 (31.0)	240 (29.2)	237 (29.5)
2009–2010	1135(34.4)	299 (35.5)	272 (32.9)	276 (33.6)	288 (35.9)
2011–2012	1193(36.2)	310 (36.8)	299 (36.2)	306 (37.2)	278 (34.6)
Macrosomia (%)	302(9.1)	–	–	–	303 (37.9)
FEV_1_(% predicted) (mean ± SD)	98.52 (14.62)	95.57 (13.95)	98.73 (13.61)	99.38 (13.51)	100.48 (13.54)
FVC (% predicted) (mean ± SD)	98.97 (13.78)	97.66 (13.22)	100.23 (13.57)	100.89 (12.66)	101.77 (12.93)
FEV_1_/FVC(% predicted) (mean ± SD)	98.76 (7.15)	97.33 (7.44)	98.00 (6.92)	97.89 (6.94)	98.12 (6.97)
FEF_25 − 75_(% predicted) (mean ± SD)	100.38 (29.77)	88.58 (24.57)	93.81 (24.36)	94.00 (23.78)	95.92 (25.00)

PIR, poverty income ratio; FEV_1_, forced expiratory volume in 1 s; FVC, forced vital capacity; FEF_25 − 75_, forced expiratory flow between 25% and 75% of vital capacity; SD, standard deviation.

To ensure the robustness of the results, we performed the following sensitivity analysis. (1) We rerun all models for the relationship between birth weight and lung function, limiting our analysis to participants with FEV_1_ and FVC values grade A or B. (2) We rerun all models for the association of birth weight and lung function with the percentage of predicted indicators using the NHANES criteria.

All models were adjusted for age (continuous), gender (boys/girls), height, race/ethnicity (Mexican American/Other Hispanic/Non-Hispanic White/Non-Hispanic Black/Other Race), mother's age at birth of children (continuous), PIR (≤ 1/>1), education level (≤ 5 grade/6–8 grade or 9–12 grade), asthma (yes/no), maternal smoking during pregnancy (yes/no) and survey cycles (2007–2008, 2009–2010, or 2011–2012).

All analyses were performed using R (version 4.1.0; R Development Core Team). *P*-values of < 0.05 were considered to be statistically significant.

## Results

### Participant characteristics

In total, 3,295 children (aged 6–15 years) were included in the present study. The characteristics of the study participants based on the quartiles of birth weight are shown in Table 1. Overall, the mean age (SD) of the participants was 10.48 (2.77) years and 1,692 (51.4%) were boys. 18.6% of the children reported current asthma, 9.1% were macrosomia, and 12.5% were exposed to maternal smoking during pregnancy. The mean (SD) for percentages of predicted FEV_1_, FVC, FEV_1_/FVC, and FEF_25 − 75_ were 98.52% (14.62), 98.97% (13.78), 98.76% (7.15), and 100.38% (29.77), respectively. Additionally, the range of birth weight across 1st−4th quartiles were < 2.95, 2.92–3.32, 3.32–3.66, and >3.66 kg, respectively. There was no substantial difference in demographic and lifestyle characteristics between included and excluded children (Supplementary Table 1).

### Association of birth weight with lung function

**Figure 2:**
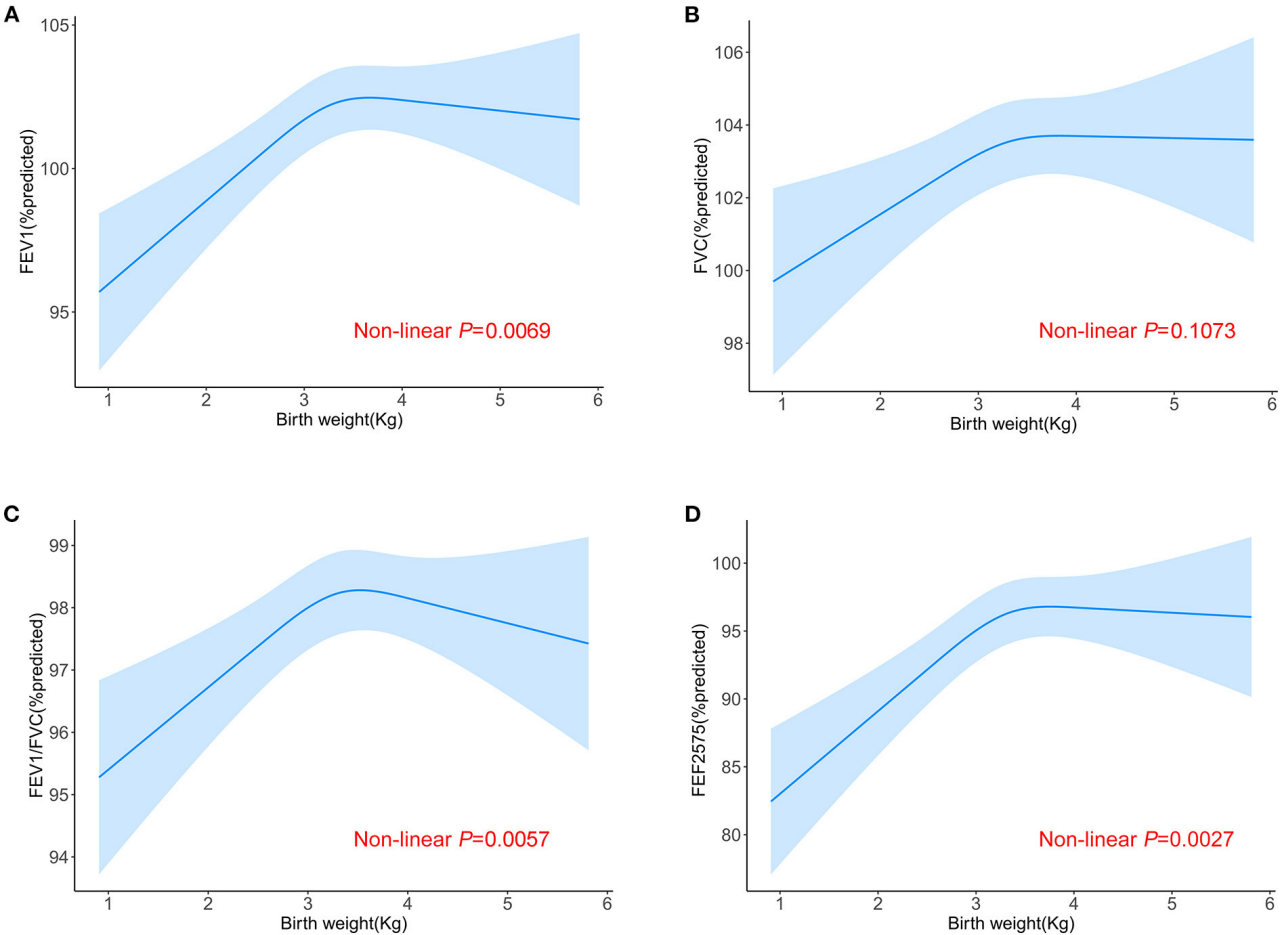
Non-linear relationship between birth weight and lung function. Relationship between birth weight and FEV_1_ %predicted **(A)**, FVC %predicted **(B)**, FEV_1_/FVC %predicted **(C)** and FEF_25 − 75_ %predicted **(D)** in all the participants aged 6–15. Adjusted for age (continuous), race/ethnicity, sex, height, PIR, education level, mother's age at birth of children, maternal smoking during pregnancy as well as NHANES cycle.

The linear associations of birth weight with lung function were summarized in Table 2. We found that birth weight, when treated as a continuous variable, was positively associated with all lung function metrics both in the unadjusted and adjusted models (all *P* for trend < 0.05). When birth weight was treated as a categorical variable, the results of FEV_1_ %predicted, FVC %predicted and FEF_25 − 75_ %predicted were similar to those seen in the continuous variable model structure (all *P* for trend < 0.05). Compared to the reference group (birth weight < 2.95 kg), the positive association between birth weight and FEV_1_/FVC %predicted was only found in the fourth quantile of birth weight (>3.6 kg). The linear trend was also found between birth weight and FEV_1_/FVC %predicted although this was not statistically significant in the adjusted model (*P* for trend = 0.253). Then, we conducted a generalized additive model to test non-linearity using penalized smoothing regression splines with a degree of 3. The fully adjusted model showed a non-linear association of birth weight with FEV_1_ %predicted, FEV_1_/FVC %predicted, and FEF_25 − 75_ %predicted (*P* for non-linearity was 0.0069, 0.0057, and 0.0027, respectively) (Figure 2). We further conducted a threshold effect analysis of birth weight on lung function, and detected the turning point for birth weight was 3.6 kg. When the birth weight was < 3.6 kg, the adjusted change in FEV_1_ %predicted per 1 kg birth weight was 2.43 (95% CI 1.36 to 3.51), in FVC %predicted was 1.80 (95% CI 0.79 to 2.82), in FEV_1_/FVC %predicted was 0.70 (95% CI 0.09 to 1.31), in FEF_25 − 75_ %predicted was 4.24(95% CI 2.20 to 6.29). However, when the birth weight was ≥3.6 kg, per 1 kg increase in birth weight was significantly associated with lower FEV_1_ %predicted (β = −1.58, 95% CI −3.58 to −0.03), lower FEV_1_/FVC %predicted (β = −1.36, 95% CI −2.48 to −0.25), and lower FEF_25 − 75_ %predicted (β = −3.99, 95% CI −7.92 to −0.06), respectively. In addition, no significant association was found between birth weight and FVC% predicted when the birth weight was ≥3.6 kg (Table 3).

**Table 2:** The linear association of birth weight (per 1 kg increase) with lung function in children aged 6–15 in the NHANES 2007–2012 survey (*n* = 3,295).

**Lung function**	**Birth weight (kg)**	**Birth weight quartiles [Median (IQR)]**				***P* for trend**
		**Q1 (< 2.95 kg)** **2.72 (2.35–2.86)**	**Q2 (2.95–3.32 kg)** **3.18 (3.06–3.23)**	**Q3 (3.32–3.66 kg)** **3.48 (3.40–3.57)**	**Q4 (>3.66 kg)** **3.91 (3.80–4.20)**	
**FEV**_**1**_ **%predicted**						
Unadjusted	3.12 (2.37, 3.86)	Reference	3.16 (1.85, 4.47)	3.81 (2.50, 5.12)	4.91 (3.59, 6.23)	< 0.001
Adjusted	1.86 (1.18, 2.54)	Reference	1.71 (0.53, 2.89)	2.27 (1.08, 3.46)	2.69 (1.47, 3.90)	< 0.001
**FVC %predicted**						
Unadjusted	2.56 (1.84, 3.27)	Reference	2.57 (1.31, 3.83)	3.23 (1.97, 4.49)	4.10 (2.84, 5.37)	< 0.001
Adjusted	1.49 (0.84, 2.14)	Reference	1.33 (0.21, 2.46)	2.03 (0.89, 3.17)	2.16 (1.00, 3.32)	< 0.001
**FEV**_**1**_ **/FVC %predicted**						
Unadjusted	0.58 (0.20, 0.97)	Reference	0.66 (−0.02, 1.34)	0.55 (−0.13, 1.23)	0.79 (0.10, 1.47)	0.040
Adjusted	0.39 (0.01, 0.77)	Reference	0.43 (−0.23, 1.08)	0.19 (−0.47, 0.86)	0.49 (−0.18, 1.18)	0.253
**FEF**_**25 − 75**_ **%predicted**						
Unadjusted	4.73 (3.40, 6.06)	Reference	5.23 (2.89, 7.57)	5.42 (3.08, 7.77)	7.35 (4.98, 9.71)	< 0.001
Adjusted	3.39 (2.09, 4.71)	Reference	3.59 (1.33, 5.85)	3.49 (1.21, 5.77)	5.04 (2.71, 7.38)	< 0.001

PIR, poverty income ratio; FEV_1_, forced expiratory volume in 1 s; FVC, forced vital capacity; FEF_25 − 75_, forced expiratory flow between 25 and 75% of vital capacity.

Unadjusted, crude model. Adjusted: adjusted for age (continuous), race/ethnicity, sex, height, PIR, education level, mother's age at birth of children, maternal smoking during pregnancy as well as NHANES cycle.

**Table 3:** Threshold effect analysis of relationship between birth weight and lung function using two-piecewise regression models in children aged 6–15 in the NHANES 2007–2012 survey (*n* = 3,295).

**Birth weight (kg)**	**Crude β (95% CI)**	***P*-value**	**Adjusted β (95% CI)**	***P*-value**
**FEV**_**1**_ **%predicted**				
< 3.6	3.71 (2.54, 4.88)	< 0.001	2.43 (1.36, 3.51)	< 0.001
≥3.6	−1.21 (−3.44, 1.01)	0.285	–1.58 (–3.58, –0.03)	0.044
Likelihood ratio test		< 0.001		< 0.001
**FVC % predicted**				
< 3.6	2.94 (1.81, 4.07)	< 0.001	1.80 (0.79, 2.82)	< 0.001
≥3.6	0.11 (−2.01, 2.23)	0.919	−0.15 (−2.08, 1.78)	0.879
Likelihood ratio test		< 0.001		< 0.001
**FEV**_**1**_ **/FVC %predicted**				
< 3.6	0.87 (0.25, 1.48)	< 0.001	0.70 (0.09, 1.31)	0.024
≥3.6	−1.26 (−2.39, −0.13)	0.029	–1.36 (–2.48, –0.25)	0.016
Likelihood ratio test		< 0.001		< 0.001
**FEF**_**25–75**_ **% predicted**				
< 3.6	5.54 (3.47, 7.61)	< 0.001	4.24 (2.20, 6.29)	< 0.001
≥3.6	−3.68 (−7.77, 0.41)	0.078	–3.99 (–7.92, –0.06)	0.046
Likelihood ratio test		< 0.001		< 0.001

PIR, poverty income ratio; FEV_1_, forced expiratory volume in 1 s; FVC, forced vital capacity; FEF_25 − 75_, forced expiratory flow between 25 and 75% of vital capacity.

Adjusted for age, race/ethnicity, sex, height, PIR, education level, mother's age at birth of children, maternal smoking during pregnancy as well as NHANES cycle.

We further performed exploratory subgroup analyses to assess the association between birth weight and lung function in two groups of participants divided at the turning point of birth weight was 3.6 kg. All subgroup variables included age (< 11 vs. ≥11 years), gender (boys vs.girls), maternal smoking during pregnancy (yes vs. no), PIR (>1 vs. ≤ 1) and asthma (yes vs. no). We did not find any evidence of effect modification for the association of birth weight and lung function by these variables (all *P* for interaction >0.05) (Supplementary Figures 1–4). Moreover, the non-linear association was robust when we restricted our analysis to participants with FEV_1_ and FVC values grade A or B, or when the percentage of predicted indicators used the NHANES criteria (Supplementary Figures 5, 6).

### Association of birth weight with the risk of asthma

**Figure 3:**
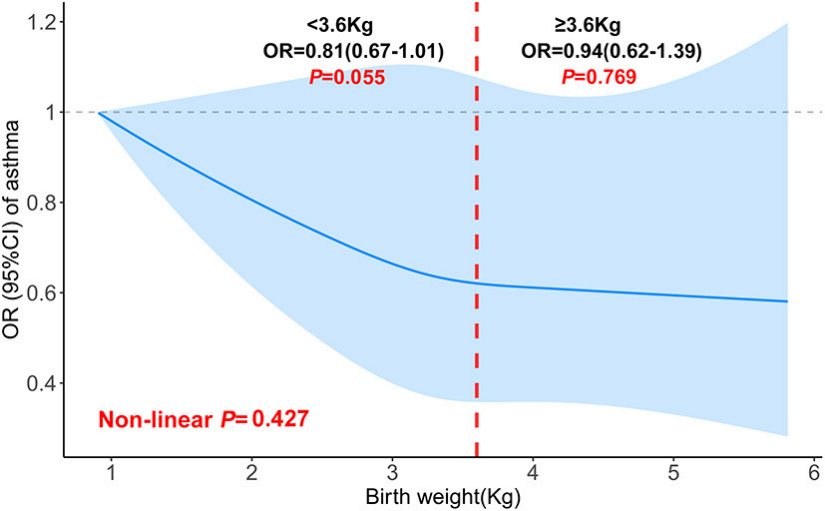
Non-linear relationship between birth weight and asthma in children aged 6–15 (the cut-off point was 3.6 kg). Adjusted for age (continuous), race/ethnicity, sex, height, PIR, education level, mother's age at birth of children, maternal smoking during pregnancy as well as NHANES cycle.

We also investigated the relationship between birth weight and the risk of asthma. When birth weight was treated as a continuous variable, no association was found whether in the crude model or in the adjusted model. After adjustment for all covariates, in comparison with the reference group (birth weight < 2.95 kg), only children in the third quartile (3.32–3.66 kg) had a 24% lower risk of asthma [OR 0.76, 95% CI (0.59–0.98)], indicating a possible non-linear association of birth weight with the risk of asthma. No significant non-linear relationship was found in the fully adjusted generalized additive model (Table 4). However, we could also observe some differences when birth weight was divided at 3.6 kg. The increase in birth weight was borderline significant with the risk of asthma [OR 0.81, 95% CI (0.67–1.01)] when birth weight was < 3.6, and no association was found when birth weight was ≥3.6 kg (Figure 3).

**Table 4:** The linear association between birth weight and asthma in children aged 6–15 in the NHANES 2007–2012 survey (*n* = 3,295).

**Birth weight (kg)**	**Cases/Total(*n*)**	**Asthma**			
		**Crude OR (95% CI)**	***P* value**	**Adjusted OR (95% CI)**	***P* value**
**Continuous**	613/2682	0.90 (0.78, 1.03)	0.139	0.88 (0.76, 1.02)	0.075
**Quartile**					
< 2.95	167/676	Reference		Reference	
2.95–3.32	157/670	0.95 (0.74, 1.21)	0.6696	0.93 (0.73, 1.19)	0.719
3.32–3.66	133/689	0.78 (0.61, 1.00)	0.0543	0.76 (0.58, 0.98)	0.033
>3.66	156/647	0.98 (0.76, 1.24)	0.8449	0.93 (0.73, 1.20)	0.677
*P* for trend			0.499		0.305

Adjusted for age, race/ethnicity, sex, height, PIR, education level, mother's age at birth of children, maternal smoking during pregnancy as well as NHANES cycle.

## Discussion

To the best of our knowledge, this is the first study to examine the non-linear relationship between birth weight and lung function in a large and representative sample of children from the U.S. population. We observed a non-linear relationship between birth weight and lung function in school-age children. Moreover, we also found some evidence for the non-linear association of birth weight with the risk of asthma. These findings refine and extend previous knowledge about the association of birth weight with lung function, and remind us of the need for early lung function assessment not only in children with low birth weight, but also with high birth weight.

Our findings on the linear effects of birth weight on children's lung function metrics are in line with previous reports. A large meta-analysis including data from 18 cohorts with 26,069 children found strong evidence of an association between birth weight and adult FVC, with a 59.4 mL (95% CI: 43.3–75.5) higher FVC in adulthood per 1 kg increase in birth weight (18). Higher birth weight was also associated with greater FEV_1_, FVC and FEF_25 − 75_ in children at age 10 and 18 years (19). Interestingly, other than these indexes, our current analysis also found the linear relationship between birth weight and FEV_1_/FVC %predicted, although only in the unadjusted model. More studies are needed to confirm this finding.

It can be seen that earlier studies only investigated the linear relationship between birth weight and lung function. However, it is not appropriate to use a generalized linear model to analyze the correlation once the non-linear relationship is identified. For the first time, we systematically assessed the non-linear association of birth weight with lung function metrics using smoothing curve analysis and stratified analysis. We found an interesting phenomenon that the relationship between birth weight and FEV_1_ %predicted, FEV_1_/FVC %predicted, and FEF_25 − 75_ %predicted both present a non-linear function, with the positive association when the birth weight is < 3.6 kg and the negative association when the birth weight is ≥3.6 kg. The World Health Organization defined the cutoff of low birth weight as < 2.5 kg, which is based on the epidemiological evidence that infants with birth weight < 2.5 kg had a higher mortality risk than ≥2.5 kg (20). However, it may not be the optimal threshold for the determination of respiratory health. Consistent with our results, a recent study suggested that birth weight of between 3.43 and 3.80 kg might represent a potential threshold for reducing hypertension risk (21). Also, the combined available evidence from observational studies supports that the risk of childhood acute myeloid leukemia may be elevated at both high and low extremes of birth weight (22). Therefore, previous knowledge about the linear relationship between birth weight and lung function should be re-examined, and more information about the effects of birth weight is worth exploring further in the future.

Our findings showed that higher birth weight is associated with airflow obstruction and small airway dysfunction, indicated by a decrease in FEV_1_/FVC %predicted and FEF_25 − 75_ %predicted with an increase in birth weight (≥3.6 kg). In addition, the non-linear trend between birth weight and childhood asthma also existed, although it is not statistically significant. Several studies have illustrated the effects of birth weight on the risk of asthma, but the results are inconsistent. A retrospective cohort study found that low birth weight is not associated with asthma in childhood in the absence of smoking in pregnancy (23). However, meta-analysis results suggested that low birth weight is associated with an increased risk of asthma both in children and adults, and high birth weight was not associated with an increased risk of asthma (11, 24). Similarly, another study showed that low birth weight is a risk factor for asthma independently of gestational age, sex, birth length and Apgar score. Taken together, the observation that asthma is associated with both increased and reduced birth weight suggested a complex underlying mechanism. In the current analysis, our results showed that only children in the third quartile (3.32–3.66 kg) had a 24% lower risk of asthma when compared with the reference group (birth weight < 2.95 kg). Furthermore, our findings also indicated a possible inflection point for birth weight was 3.6 kg, which might represent a potential threshold for reducing asthma risk. Diabetes during pregnancy, maternal obesity and asthma are also important factors for the association between birth weight and children asthma (25, 26), however, these factors are unavailable in our analysis. Therefore, we should not overinterpret the results, future studies are warranted to verify this result.

The biological mechanisms underlying birth weight affecting lung function are complex and remain equivocal. There is an indication that birth weight, as a prenatal or early post-natal environment, may impact lung function in childhood by influencing the lung structure and development, which may have potential implications for later chronic lung disease (8). Moreover, exposures that result in retarded fetal growth, including low birth weight, may irrecoverably constrain the growth of an individual's airways and result in poorer lung function that persists into adulthood (27). Beyond lung structure, low birth weight also has implications for immunocompetence and increasing vulnerability to insults in later life (28). Animal studies also found that low birth weight could disrupt lung angiogenic signaling, elastic fiber formation, and microvascular formation in a time-dependent manner in an experimental rat model (29). On the other hand, some potential explanations have been suggested for the association between high birth weight and respiratory health. The first explanation relates to lung mechanics, such as the disadvantage of respiratory muscle function because of obesity. Second, obesity may influence immune development, altering the risk of developing chronic asthma (30).

The strength of the present study benefits from the large and nationally representative population sample, which rendered our findings generalizable. However, several limitations also need to be noted. First of all, the birth weight was recalled by an “adult proxy” which could be subject to recall bias, nonetheless, there is evidence that only 1.6% of birth weight might have been misclassified into low, normal or high birth weight (31). Second, our analysis only included smoking during pregnancy and the mother's age at birth of children as potential maternal confounders, and data on other maternal factors (such as gestation age, diabetes, lung disease, and maternal diet) were not available in the NHANES database. This and other potential confounders such as allergic sensitization, use of asthma mediation, and exposure to air pollution (32, 33) could have biased the results presented. Last, our present results were only limited to U.S. children, so the findings still need to be confirmed in other populations.

In summary, although not all maternal factors were accounted for, we reported for the first time a non-linear association between birth weight and lung function, and we determined an inflection point for birth weight among 6–15 years old children. Moreover, our results also raised the possibility of a non-linear association of birth weight with the risk of asthma. Given the development of lung function may be affected by hypogenesis *in utero*, further studies are warranted to verify our results and explore underlying mechanisms.

## Data availability statement

The datasets presented in this study can be found in online repositories. The names of the repository/repositories and accession number(s) can be found below: https://www.cdc.gov/nchs/nhanes/index.htm.

## Ethics statement

The studies involving human participants were reviewed and approved by data collection for NHANES was approved by the NCHS Research Ethics Review Board. All participants provided written informed consent. Written informed consent to participate in this study was provided by the participants' legal guardian/next of kin.

## Author contributions

MY performed the statistical analyses, interpreted the data, and drafted the manuscript. HM, JD, and LY contributed to the data acquisition and review for important intellectual content. LH and LY revised the manuscript. HX conceived and designed this study and final version approval. All authors have read and approved the final manuscript.

## Funding

This study was supported by Natural Science Foundation of Hubei Province of China (No. 2021CFB176).

## Conflict of interest

The authors declare that the research was conducted in the absence of any commercial or financial relationships that could be construed as a potential conflict of interest.

## Publisher's note

All claims expressed in this article are solely those of the authors and do not necessarily represent those of their affiliated organizations, or those of the publisher, the editors and the reviewers. Any product that may be evaluated in this article, or claim that may be made by its manufacturer, is not guaranteed or endorsed by the publisher.
