# Supplementary material for: Non-linear association of birth weight with lung function and risk of asthma: A population-based study
Source: Front Public Health. 2022 Nov 24;10:999602. doi: 10.3389/fpubh.2022.999602 (PMC9731215; doi:10.3389/fpubh.2022.999602)
Supplement: Supplementary file 1 [file Data_Sheet_1.pdf]

Supplementary Materials

Nonlinear association of birth weight with lung function and risk of asthma: a population-based study

Meng Yang[1#], Hong Mei[1], Juan Du[1], Linling Yu[2], Liqin Hu[1], Han Xiao[1]*

[1]Institute of Maternal and Child Health, Wuhan Children's Hospital (Wuhan Maternal and Child Health care Hospital), Tongji Medical College, Huazhong University of Science and Technology, Wuhan, China.

[2]Department of Occupational and Environmental Health, School of Public Health, Tongji Medical College, Huazhong University of Science and Technology, Wuhan, Hubei 430030, China.

*Corresponding author:

Dr. Han Xiao

Wuhan Children's Hospital (Wuhan Maternal and Child Healthcare Hospital), Tongji Medical College, Huazhong University of Science and Technology, Wuhan 430000, China

E-mail：tjxiaohan1980@163.com

Table S1 Characteristics of included children and those excluded for study participants in NHANES 2007-2012.

Characteristics	Included	Excluded	*P*
No. subjects	3295	1228	
Age, year (mean ± SD)	10.48 (2.77)	9.95 (2.72)	<0.001
Race/ethnicity (%)			0.014
Mexican American	823 (25.0)	302 (24.6)	
Other Hispanic	389 (11.8)	153 (12.5)	
Non-Hispanic White	986 (29.9)	324 (26.4)	
Non-Hispanic Black	797 (24.2)	300 (24.4)	
Other Race - Including Multi-Racial	300 (9.1)	149 (12.1)	
Gender (%)			0.032
Boys	1692 (51.4)	586 (47.7)	
Girls	1603 (48.6)	642 (52.3)	
Education levels (%)			<0.001
≤5 grade	2150 (65.3)	883 (72.0)	
6-8 grade	911 (27.6)	273 (22.3)	
9-12 grade, No Diploma	234 (7.1)	70 (5.7)	
Maternal smoking during pregnancy (%)			0.425
Yes	412 (12.5)	142 (11.6)	
No	2883 (87.5)	1085 (88.4)	
Poverty index ratio (PIR) (%)			0.247
PIR≤1	1106 (33.6)	284 (31.5)	
PIR>1	2189 (66.4)	619 (68.5)	
Asthma (%)			0.320
Yes	613 (18.6)	212 (17.3)	
No	2682 (81.4)	1016 (82.7)	
NHANES cycles			<0.001
2007-2008	967 (29.3)	448 (36.5)	
2009-2010	1135 (34.4)	370 (30.1)	
2011-2012	1193 (36.2)	410 (33.4)	

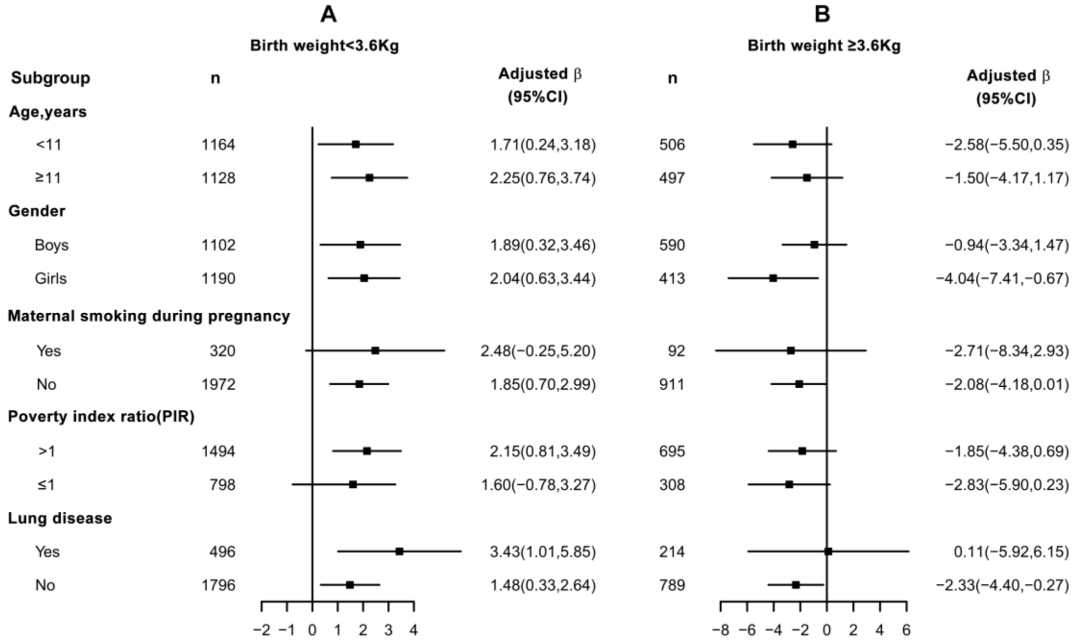

Figure S1. Stratified analysis for the relationship between birth weight and FEV_1% predicted in various subgroups divided at 3.6kg in all participants. Adjusted for age, race/ethnicity, sex, height, PIR, education level, mother's age at birth of children, maternal smoking during pregnancy as well as NHANES cycle.

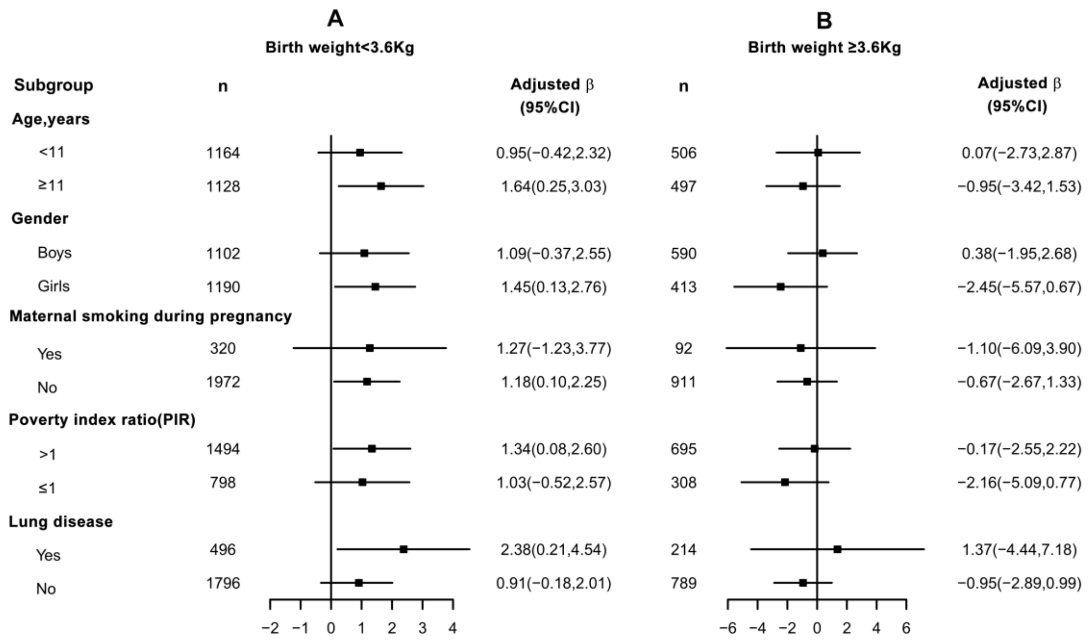

Figure S2. Stratified analysis for the relationship between birth weight and FVC% predicted in various subgroups (**A** birth weight<3.6kg, **B** birth weight≥3.6kg) in children. Adjusted for age, race/ethnicity, sex, PIR, education level, mother's age at birth of children, maternal smoking during pregnancy as well as NHANES cycle.

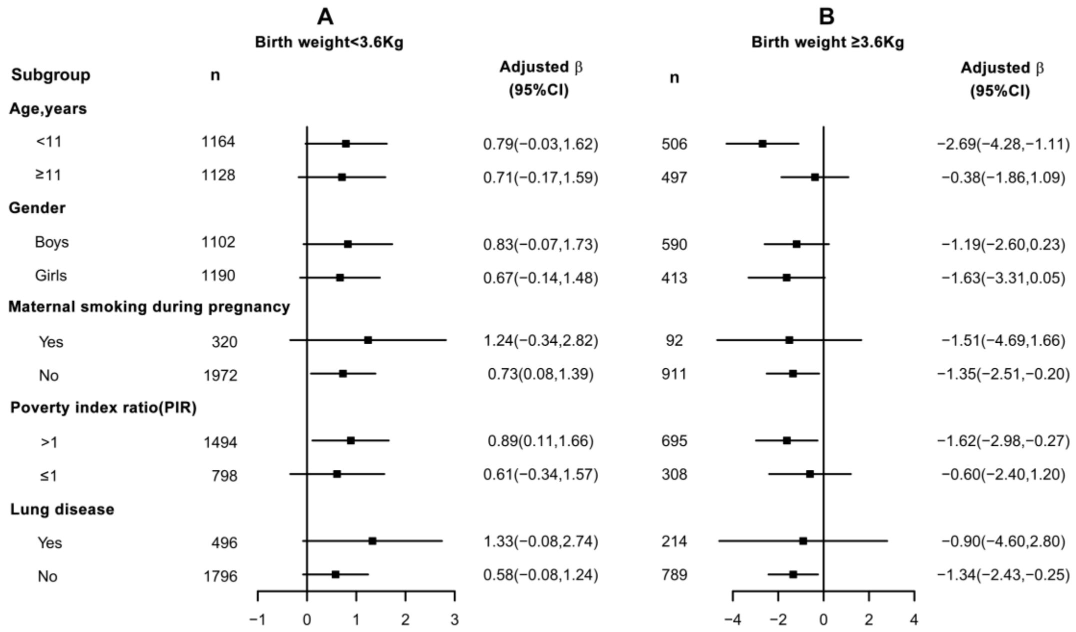

Figure S3. Stratified analysis for the relationship between birth weight and FEV_1/FVC % predicted in various subgroups (**A** birth weight<3.6kg, **B** birth weight≥3.6kg) in children. Adjusted for age, race/ethnicity, sex, PIR, education level, mother's age at birth of children, maternal smoking during pregnancy as well as NHANES cycle.

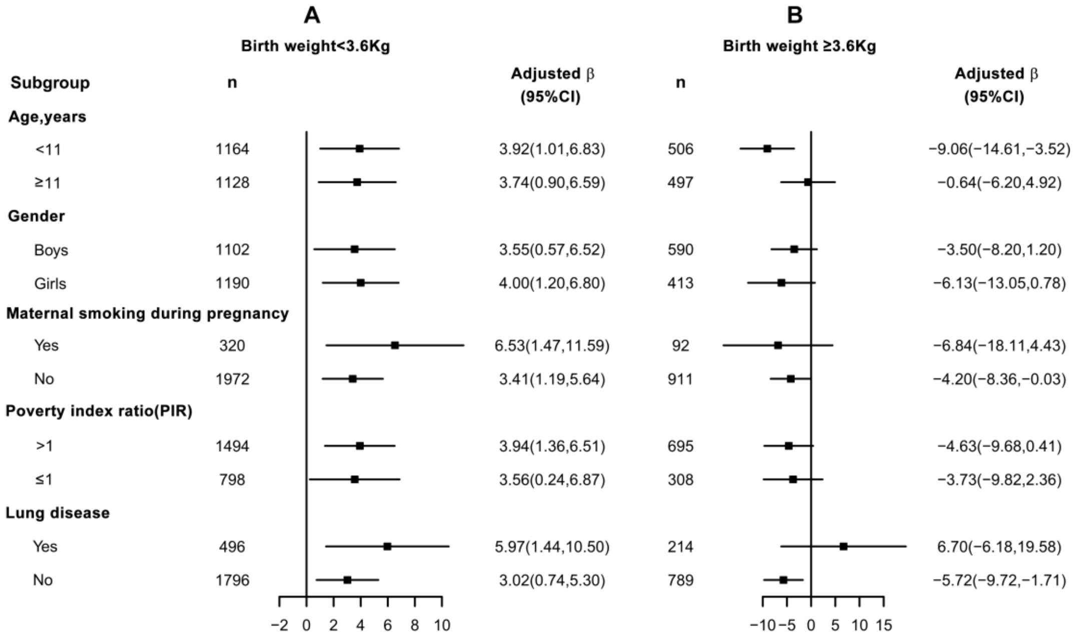

Figure S4. Stratified analysis for the relationship between birth weight and FEF_{25-75} %predicted in various subgroups (**A** birth weight<3.6kg, **B** birth weight≥3.6kg) in children. Adjusted for age, race/ethnicity, sex, PIR, education level, mother's age at birth of children, maternal smoking during pregnancy as well as NHANES cycle.

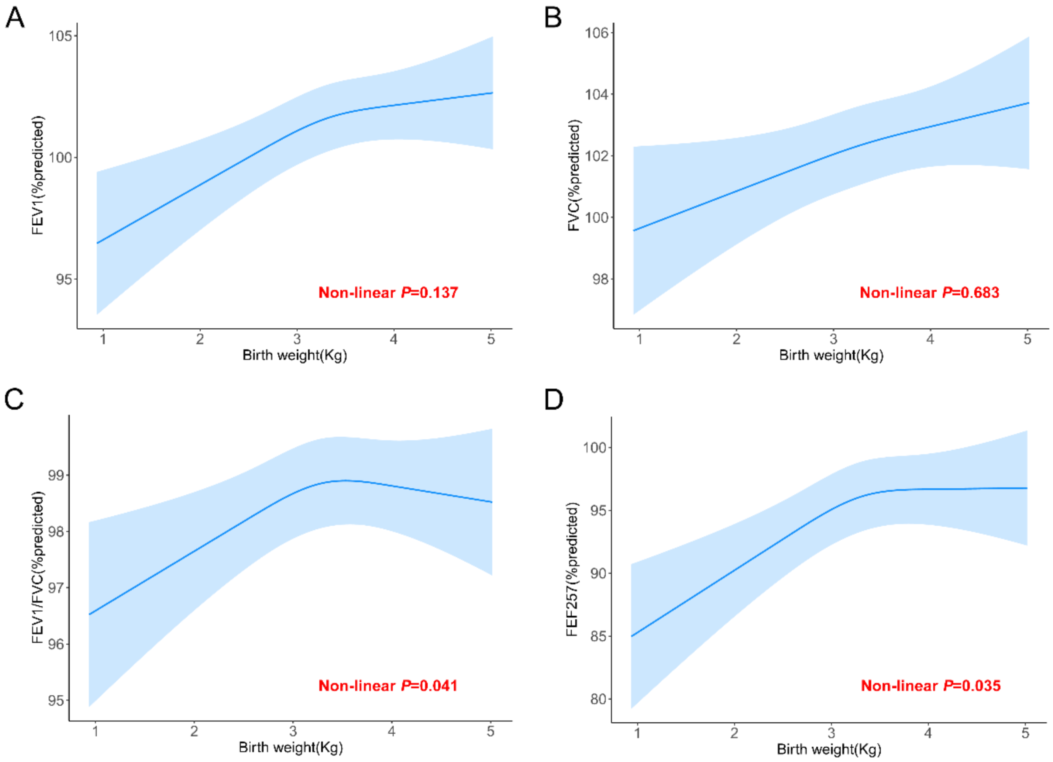

Figure S5. Nonlinear relationship between birth weight and lung function when restricted our analysis to participants with FEV_1 and FVC values grade A or B. Relationship between birth weight and FEV_1% predicted (A), FVC% predicted (B), FEV_1/FVC % predicted (C) and FEF_{25-75} % predicted (D) in children. Adjusted for age, race/ethnicity, sex, height, PIR, education level, mother's age at birth of children, maternal smoking during pregnancy as well as NHANES cycle.

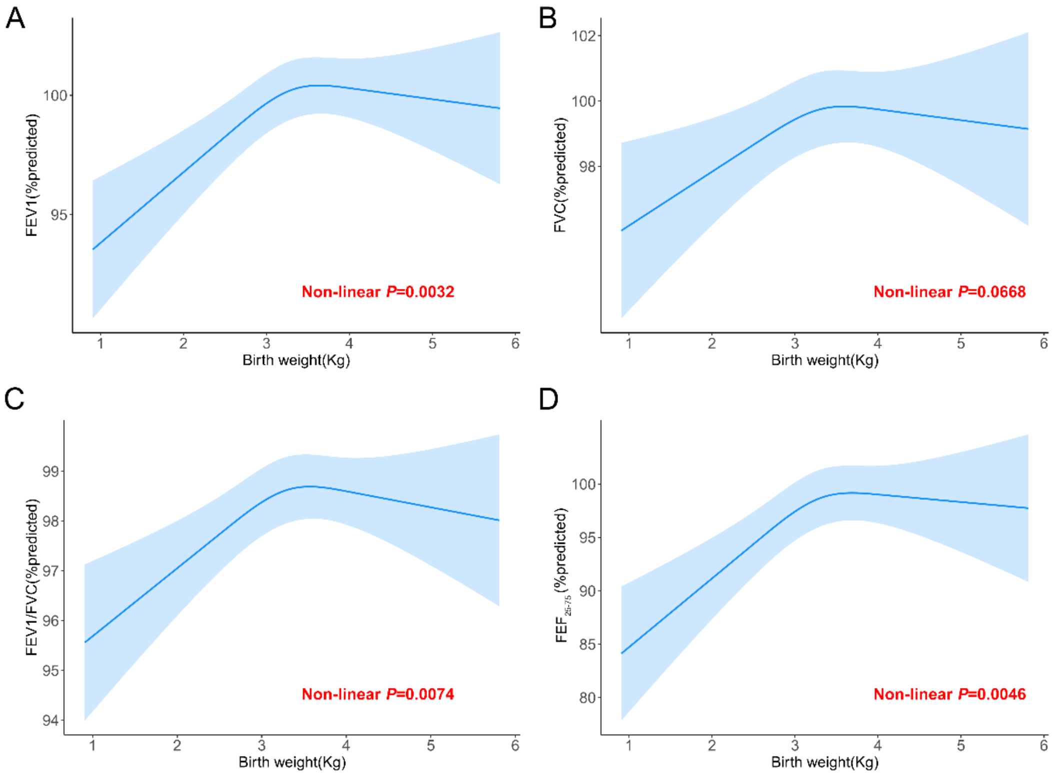

Figure S6. Nonlinear relationship between birth weight and lung function when the percentage of predicted indicators used the NHANES criteria. Relationship between birth weight and FEV_1% predicted (A), FVC% predicted (B), FEV_1/FVC % predicted (C) and $FEF_{25\text{-}75}$ % predicted (D) in children. Adjusted for age, race/ethnicity, sex, height, PIR, education level, mother's age at birth of children maternal smoking during pregnancy as well as NHANES cycle.
